# Nomogram for predicting the 28-day mortality risk of patients with subendocardial infarction

**DOI:** 10.3389/fcvm.2025.1459855

**Published:** 2025-06-13

**Authors:** Menglei Li, Beiping Song, Xianjing Zeng, Xunguo Wang, Ao Ma, Zhichao Meng, Jiehao Zhu, Xiubao Song, Xianwu Lan, Minghui Tan

**Affiliations:** ^1^ Department of Orthopedics, The First Affiliated Hospital of Jinan University, Guangzhou, China; ^2^ College of Life Science and Technology, Jinan University, Guangzhou, China; ^3^ Department of Nuclear Medicine, Hunan Provincial People’s Hospital (The First Affilated Hospital of Hunan Normal University), Changsha, Hunan, China; ^4^ Department of Rehabilitation Medicine, The First Affiliated Hospital of Jinan University, Guangzhou, China; ^5^ Department of Cardiology, The First Affiliated Hospital of Jinan University, Guangzhou, China

**Keywords:** subendocardial infarction, nomogram, MIMIC-III database, prognosis, mortality

## Abstract

**Background:**

Subendocardial myocardial infarction (SEMI) represents a more severe form of myocardial infarction. Currently, there lacks a comprehensive clinical index for predicting mortality in cases of subendocardial myocardial infarction. The objective of our study was to develop and evaluate a nomogram for predicting the 28-day risk of mortality among patients with SEMI.

**Methods:**

Patients diagnosed with subendocardial infarction were identified from the MIMIC-III database based on ICD-9 codes. Independent risk factors were screened utilizing the least absolute shrinkage and selection operator (LASSO) method alongside multivariate logistic regression. These identified risk factors were then employed to construct a nomogram aimed at predicting the 28-day mortality risk in patients with subendocardial infarction. The performance of the nomogram was evaluated by the Area Under the Curve (AUC), calibration curves, Hosmer-Lemeshow test, Integrated Discrimination Improvement (IDI), Net Reclassification Improvement (NRI), Decision Curve Analysis (DCA).

**Results:**

A total of 3046 patients with subendocardial infarction were included in the study. Logistic regression analysis revealed that age, GCS score, creatinine level, hematocrit, hemoglobin, international normalized ratio, blood urea nitrogen level, urine output, heart rate, respiratory rate, peripheral oxygen saturation, peripheral vascular disease, diabetes complications, and solid tumors were independent risk factors for 28-day mortality. The AUC values of the nomogram surpassed those of the Acute Physiology Score III (APSIII), Simplified Acute Physiology Score II (SAPSII), and Sequential Organ Failure Assessment (SOFA) scoring systems in both the training and validation cohorts. Calculation of the IDI and NRI, along with DCA analysis, indicated a greater net benefit of the nomogram model.

**Conclusion:**

This study successfully identified independent risk factors for 28-day mortality in patients with SEMI. A nomogram model was developed to predict mortality, offering potential assistance in improving the prognosis of SEMI patients.

## Introduction

Subendocardial infarction (SEMI) is characterized by widespread coronary narrowing, fibrinous pericarditis, and recurrences or extensions often involving superjacent or adjacent areas (1). SEMI primarily results from a sudden decrease in blood flow to the myocardial tissue, leading to ischemia and subsequent tissue death (2). The most common underlying mechanism is myocardial ischemia and metabolic disturbances due to coronary vascular thrombotic occlusion resulting from rupture of a vulnerable plaque (3). This ischemia precipitates a rapid decline in cardiac contractile function, leading to the progression of cardiomyocyte death from the subendocardium to the subepicardium (2).

Physicians and scientists have extensively investigated various therapeutic interventions for subendocardial infarction (4–6). Studies indicate that percutaneous transluminal coronary angioplasty (4), intracoronary infusion of bone marrow-derived cells (BMCs) (5), and aggressive treatment strategies including revascularization and mechanical support can improve survival rates and clinical outcomes in patients with subendocardial infarction (6). However, certain comorbidities, such as advanced age, diabetes mellitus, and preexisting cardiovascular diseases, are associated with poorer prognoses (7, 8). Furthermore, patients with SEMI face a significantly higher risk of recurrent infarction, necessitating continuous monitoring and long-term management (9). Identifying prognostic risk factors is therefore essential for improving clinical decision-making and optimizing patient outcomes.

Nomograms, which serve as graphical representations of statistical models, have demonstrated significant utility in estimating individualized risks based on patient-specific and disease-related variables (10). Nomograms can be used to generate individual probabilities of clinical events, aiding clinical decision-making and enabling the development of personalized medicine (10, 11). Prior studies have validated the effectiveness of nomograms in predicting mortality for conditions such as intracerebral hemorrhage (12), alcohol-related cirrhosis (13), and bone metastasis of pancreatic cancer (14), which are anticipated to enhance the prognosis of associated diseases. However, the nomogram for predicting the risk factors regarding mortality in patients with subendocardial myocardial infarction has received little attention. In this study, we have developed a nomogram based on a large cohort from the MIMIC-III database to predict the 28-day risk of mortality in patients with subendocardial myocardial infarction. This nomogram aims to improve early risk stratification and assist clinicians in optimizing patient management strategies.

## Materials and methods

### Data source

All data utilized in this study were obtained from the MIMIC-III database, which integrates de-identified comprehensive clinical data on patients admitted to Beth Israel Deaconess Medical Center, Boston, Massachusetts, encompassing 53,423 cases of adult patients (16 years of age or older) admitted to intensive care units of various hospitals between 2001 and 2012 (15). Detailed information regarding the clinical care of patients in this database is anonymized, obviating the need for informed consent for this study. Access to the MIMIC-III database was granted to the researcher after completing a series of courses provided by the National Institutes of Health, along with the requisite relevant assessments (a credentialed user on PhysioNet; certificate number 40269495).

### Patients and variables

**Figure 1:**
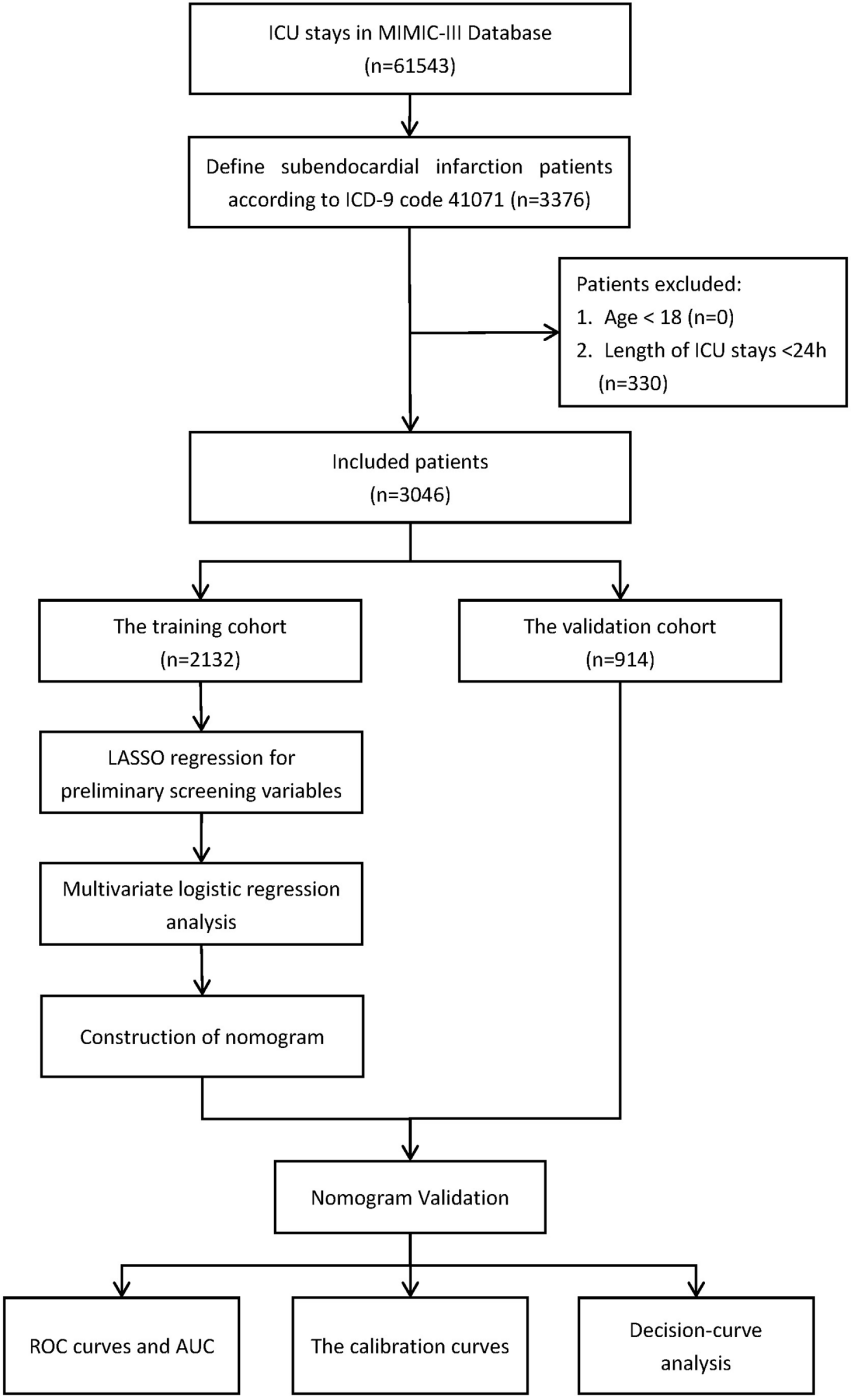
Flow diagram of the study.

For this study, the necessary data were extracted using structured query language in Navicat Premium (version 11.2.7.0). Patients diagnosed with subendocardial infarction were extracted from the MIMIC-III database using ICD-9 code 410.7 (relative to I21.4 in ICD-10). The exclusion criteria were as follows (1) age less than 18 years and (2) those who were treated in the ICU for less than 24 h. The flow chart of the study process is depicted in Figure 1.

The corresponding clinical data were obtained utilizing “icustay_id” parameters, including age, gender, race, marital status, length of ICU stays, laboratory test indicators, ICU severity score, vital signs, urine output, comorbidities, etc. Laboratory test indexes encompass anion gap, bicarbonate, creatinine, chloride, glucose, hematocrit, hemoglobin, platelet count, potassium levels, partial thromboplastin time (PTT), international normalized ratio (INR), prothrombin time (PT), sodium levels, blood urea nitrogen (BUN) level, and white blood cell (WBC) count. The vital signs used include heart rate, blood pressure, respiratory rate, temperature, peripheral oxygen saturation, glucose level, and urine output. These parameters represent the average values measured within 24 h prior to admission to the ICU. The severity scoring system employed comprises the Glasgow Coma Scale (GCS), Simplified Acute Physiology Score II (SAPS II), Sequential Organ Failure Assessment (SOFA), and Acute Physiology Score III (APS III). Comorbidities considered include heart failure, valvular disease, pulmonary circulation disorders, peripheral vascular disease, hypertension, chronic lung disease, uncomplicated diabetes mellitus, complicated diabetes mellitus, hypothyroidism, liver disease, metastatic cancer, solid tumors, rheumatoid arthritis and coagulopathy.

The primary endpoint was 28-day all-cause mortality in patients with subendocardial infarction admitted to the ICU, with patients who survived to hospital discharge designated as survivors.

### Statistical analysis

R (version 4.0.3) software was used for data cleaning, statistical analysis and graphing. Following data extraction from the MIMIC-III database, the initial step involved preprocessing, wherein patient data was screened based on exclusion criteria. Multiple imputation was then conducted for missing data, whereby subjects with missing values were assigned valid values randomly from other subjects of the same sex and age group to prevent loss of data (8). Indicators with more than 20% missing values were excluded. Subsequently, patients with subendocardial infarction were randomly allocated into training and validation sets in a 7:3 ratio. The training set facilitated variable screening, nomogram construction, and internal validation, while the validation set enabled external validation of the outcomes derived from the training set. Categorical variables were expressed as frequencies or percentages, and group comparisons were conducted using the chi-square test or Fisher's exact probability test. Continuous variables were assessed for normal distribution using the Shapiro–Wilk test. Those that followed a normal distribution were presented as mean and standard deviation and compared using the *t*-test or Mann–Whitney *U*-test. Variables not adhering to normal distribution were described using median and interquartile range. Statistical significance was set at *P* *<* *0.05*.

Logistic regression was conducted to identify key indicators influencing adverse outcomes within 28 days among patients with subendocardial infarction. Initially, Least Absolute Shrinkage and Selection Operator (LASSO) regression analysis was applied to identify potential risk factors by screening patient characteristics and clinical outcomes in the training cohort. LASSO analysis, a regression technique for variable selection and regularization, eliminates insignificant variables by penalizing regression coefficients based on their magnitude. This process enhances predictive accuracy and interpretability by shrinking coefficient estimates towards the centroid, with the degree of shrinkage determined by the parameter lambda (16). We utilized cross-validation to estimate different AUC values within the lambda range and selected the maximum lambda value where the cross-validation error remained within double the standard error of the minimum value. Subsequently, multivariate logistic regression was employed to analyze the variables selected by LASSO regression, aiming to identify significant independent prognostic factors. The results were expressed as odds ratios (OR) and 95% confidence intervals (CI). Based on these findings, a nomogram was constructed based on the identified independent risk factors, which was then utilized to predict 28-day mortality in patients with subendocardial infarction.

To evaluate the model's discriminative ability, we employed various indicators for internal and external validation. Harrell's concordance index (C-index) was computed to assess the model's predictive performance, evaluating the consistency between predicted relative hazards and observed outcomes across all subject pairs (17). Receiver operating characteristic (ROC) curves were generated using the survival_ROC package in R to analyze the accuracy of each variable in predicting mortality. Additionally, we compared the area under the curve (AUC) of our model with that of the APSIII, SAPSII, and SOFA scoring systems, with larger AUC values indicating more precise prognostic stratification (8). Statistical significance of AUC improvement was calculated by Delong's test. Through ROC curve analysis, we determined the optimal cutoff point, sensitivity, and specificity based on the Youden index. Furthermore, integrated discrimination improvement (IDI) and net reclassification improvement (NRI) were applied to assess the model's ability to predict 28-day mortality in patients with subendocardial infarction. Calibration curves were used to evaluate the agreement between model-predicted survival probabilities and observed adverse outcomes, while calibration of the nomogram was assessed using the Hosmer-Lemeshow test. The clinical validity of the model was confirmed by comparing nomograms with age scores based on decision curve analysis (DCA), enabling the calculation of net profit across a range of threshold probabilities.

## Results

### Baseline characteristics

After applying the selection and exclusion criteria, 3046 patients with subendocardial infarction were chosen from the MIMIC-III database, comprising 2132 in the training group and 914 in the validation group, respectively. The length of ICU stay (3.14 [1.96, 5.77] days vs. 3.11 [1.84, 5.76] days, *P* = 0.442), and the length of admission (9.74 [5.94, 15.8] days vs. 9.84 [5.99, 15.6] days, *P* = 0.728) were not significantly differ between the two groups. Men accounted for 58.1% and 57.8% of patients with subendocardial infarction in the training and validation groups, respectively. The median age of patients in the training and validation groups was 74.0 [63.5, 81.7] and 73.4 [64.0, 82.4] years, respectively. The percentage of patients with pulmonary circulation issues was 0.33% in both the training and validation groups. Peripheral vascular disease was present in 2.11% and 2.30% of patients in the training and validation groups, respectively, while solid tumors were present in 0.94% and 0.33% of patients. The median APSIII scores for the training and validation groups were 45.0 [34.0, 57.0] and 44.0 [33.0, 59.0], respectively. Mean blood creatinine levels (mg/dl) were 1.17 [0.83, 1.90] and 1.15 [0.85, 1.80] in the training and validation groups, while white blood cell counts (K/µl) were 11.7 [9.00, 15.2] and 11.6 [8.95, 15.4], respectively. The overall 28-day mortality rate for the selected patients was 15.2%. Additional baseline information is presented in Table 1.

**Table 1:** Characteristic of patients with subendocardial infarction.

Variables	Overall	Training cohort	Validation cohort	*P*
*N*	3,046	2,132	914	
Age (year)	73.8 [63.7,81.9]	74.0 [63.5,81.7]	73.4 [64.0,82.4]	0.602
Length of Admission (day)	9.78 [5.95,15.7]	9.74 [5.94,15.8]	9.84 [5.99,15.6]	0.728
Length of ICU stays (day)	3.13 [1.92,5.77]	3.14 [1.96,5.77]	3.11 [1.84,5.76]	0.442
Gender (%)				0.891
Female	1,279 (42.0)	893 (41.9)	386 (42.2)	
Male	1,767 (58.0)	1,239 (58.1)	528 (57.8)	
Ethnicity (%)				0.022
Black	164 (5.38)	102 (4.78)	62 (6.78)	
White	2,279 (74.8)	1,586 (74.4)	693 (75.8)	
Yellow	53 (1.74)	42 (1.97)	11 (1.20)	
Other	550 (18.1)	402 (18.9)	148 (16.2)	
Marital (%)				0.898
DSW	821 (27.0)	569 (26.7)	252 (27.6)	
Married	1,604 (52.7)	1,132 (53.1)	472 (51.6)	
Single	458 (15.0)	319 (15.0)	139 (15.2)	
Unknown	163 (5.35)	112 (5.25)	51 (5.58)	
Laboratory test				
Anion gap (mmol/L)	14.3 [12.0,17.0]	14.3 [12.0,17.0]	14.5 [12.4,16.7]	0.471
Bicarbonate (mmol/L)	23.5 [21.0,26.0]	23.5 [21.0,26.0]	23.3 [20.5,26.0]	0.126
Creatinine (mg/dl)	1.17 [0.85,1.87]	1.17 [0.83,1.90]	1.15 [0.85,1.80]	0.984
Chloride (mmol/L)	105 [101,108]	105 [101,108]	105 [102,108]	0.123
Glucose (mg/dl)	141 [120,174]	140 [120,173]	143 [121,175]	0.207
Hematocrit (g/dl)	30.9 [28.2,34.5]	30.9 [28.3,34.4]	31.0 [28.2,34.6]	0.736
Hemoglobin (g/dl)	10.4 [9.43,11.7]	10.4 [9.45,11.7]	10.5 [9.40,11.7]	0.741
Platelet (K/µl)	209 [160,269]	209 [161,268]	210 [158,271]	0.416
Potassium (mmol/L)	4.22 [3.90,4.57]	4.23 [3.90,4.58]	4.20 [3.91,4.55]	0.522
PTT (s)	38.2 [29.9,55.7]	38.2 [29.8,56.2]	38.6 [29.9,54.2]	0.752
INR	1.30 [1.15,1.50]	1.30 [1.15,1.50]	1.30 [1.18,1.50]	0.031
PT (s)	14.3 [13.3,15.8]	14.2 [13.2,15.8]	14.4 [13.3,15.9]	0.041
Sodium (mmol/L)	138 [136,140]	138 [136,140]	138 [136,140]	0.791
BUN (mmol/L)	25.0 [16.5,41.0]	25.0 [16.5,41.0]	25.7 [16.5,41.5]	0.407
WBC (K/µl)	11.7 [8.95,15.2]	11.7 [9.00,15.2]	11.6 [8.95,15.4]	0.914
Urine Output (mL)	1,645 [1,002,2,562]	1,652 [1,025,2,591]	1,611 [945,2,475]	0.180
Vital signs				
Heartrate (bpm)	83.0 [73.7,92.6]	82.8 [74.0,92.7]	83.5 [73.0,92.4]	0.539
Systolic BP (mmHg)	114 [105,126]	114 [105,126]	114 [106,126]	0.510
Diastolic BP (mmHg)	56.4 [50.7,62.9]	56.5 [50.5,62.9]	56.2 [51.0,62.9]	0.653
Mean BP (mmHg)	74.5 [69.1,81.4]	74.6 [69.0,81.6]	74.4 [69.2,80.8]	0.782
Respiratory rate (rpm)	18.6 [16.5,21.5]	18.6 [16.5,21.4]	18.8 [16.4,21.5]	0.651
Temperature (°C)	36.8 [36.4,37.2]	36.8 [36.4,37.2]	36.8 [36.4,37.2]	0.717
SpO_2_ (%)	97.6 [96.2,98.7]	97.5 [96.2,98.7]	97.6 [96.3,98.6]	0.781
Severe Score				
GCS	15.0 [14.0,15.0]	15.0 [14.0,15.0]	15.0 [14.0,15.0]	0.395
SAPSII	39.0 [31.0,48.0]	38.0 [31.0,48.0]	39.0 [31.0,48.0]	0.160
SOFA	4.00 [2.00,7.00]	4.00 [2.00,6.00]	4.00 [3.00,7.00]	0.202
APSIII	44.0 [34.0,58.0]	45.0 [34.0,57.0]	44.0 [33.0,59.0]	0.532
Comorbidities				
Congestive heart failure (%)				0.588
No	3,042 (99.9)	2,130 (99.9)	912 (99.8)	
Yes	4 (0.13)	2 (0.09)	2 (0.22)	
Valvular disease (%)				0.300
No	3,045 (100.0)	2,132 (100)	913 (99.9)	
Yes	1 (0.03)	0 (0.00)	1 (0.11)	
Pulmonary circulation (%)				1.000
No	3,036 (99.7)	2,125 (99.7)	911 (99.7)	
Yes	10 (0.33)	7 (0.33)	3 (0.33)	
Peripheral vascular (%)				0.850
No	2,980 (97.8)	2,087 (97.9)	893 (97.7)	
Yes	66 (2.17)	45 (2.11)	21 (2.30)	
Hypertension (%)				1.000
No	3,045 (100.0)	2,131 (100.0)	914 (100)	
	1 (0.03)	1 (0.05)	0 (0.00)	
Chronic pulmonary (%)				0.733
No	3,036 (99.7)	2,124 (99.6)	912 (99.8)	
Yes	10 (0.33)	8 (0.38)	2 (0.22)	
Diabetes uncomplicated (%)				1.000
No	3,044 (99.9)	2,130 (99.9)	914 (100)	
Yes	2 (0.07)	2 (0.09)	0 (0.00)	
Diabetes complicated (%)				0.098
No	3,035 (99.6)	2,127 (99.8)	908 (99.3)	
Yes	11 (0.36)	5 (0.23)	6 (0.66)	
Hypothyroidism (%)				0.510
No	3,044 (99.9)	2,131 (100.0)	913 (99.9)	
Yes	2 (0.07)	1 (0.05)	1 (0.11)	
Liver disease (%)				1.000
No	3,040 (99.8)	2,128 (99.8)	912 (99.8)	
Yes	6 (0.20)	4 (0.19)	2 (0.22)	
Metastatic cancer (%)				1.000
No	3,041 (99.8)	2,128 (99.8)	913 (99.9)	
Yes	5 (0.16)	4 (0.19)	1 (0.11)	
Solid tumor (%)				0.120
No	3,023 (99.2)	2,112 (99.1)	911 (99.7)	
Yes	23 (0.76)	20 (0.94)	3 (0.33)	
Rheumatoid arthritis (%)				0.300
No	3,045 (100.0)	2,132 (100)	913 (99.9)	
Yes	1 (0.03)	0 (0.00)	1 (0.11)	
Coagulopathy (%)				0.300
No	3,045 (100.0)	2,132 (100)	913 (99.9)	
Yes	1 (0.03)	0 (0.00)	1 (0.11)	
Status (%)				0.044
Survival	2,582 (84.8)	1,826 (85.6)	756 (82.7)	
Dead	464 (15.2)	306 (14.4)	158 (17.3)	

APSIII, acute physiology score III; BUN, blood urea nitrogen; bp, blood pressure; DSW, divorced/single/widow; INR, international normalized ratio; PTT, partial thromboplastin time; PT, prothrombin time; SAPSⅡ, simplified acute physiology score II; SOFA, sequential organ failure assessment; WBC, white blood cell; SpO2, peripheral oxygen saturation.

### Nomogram construction

**Figure 2:**
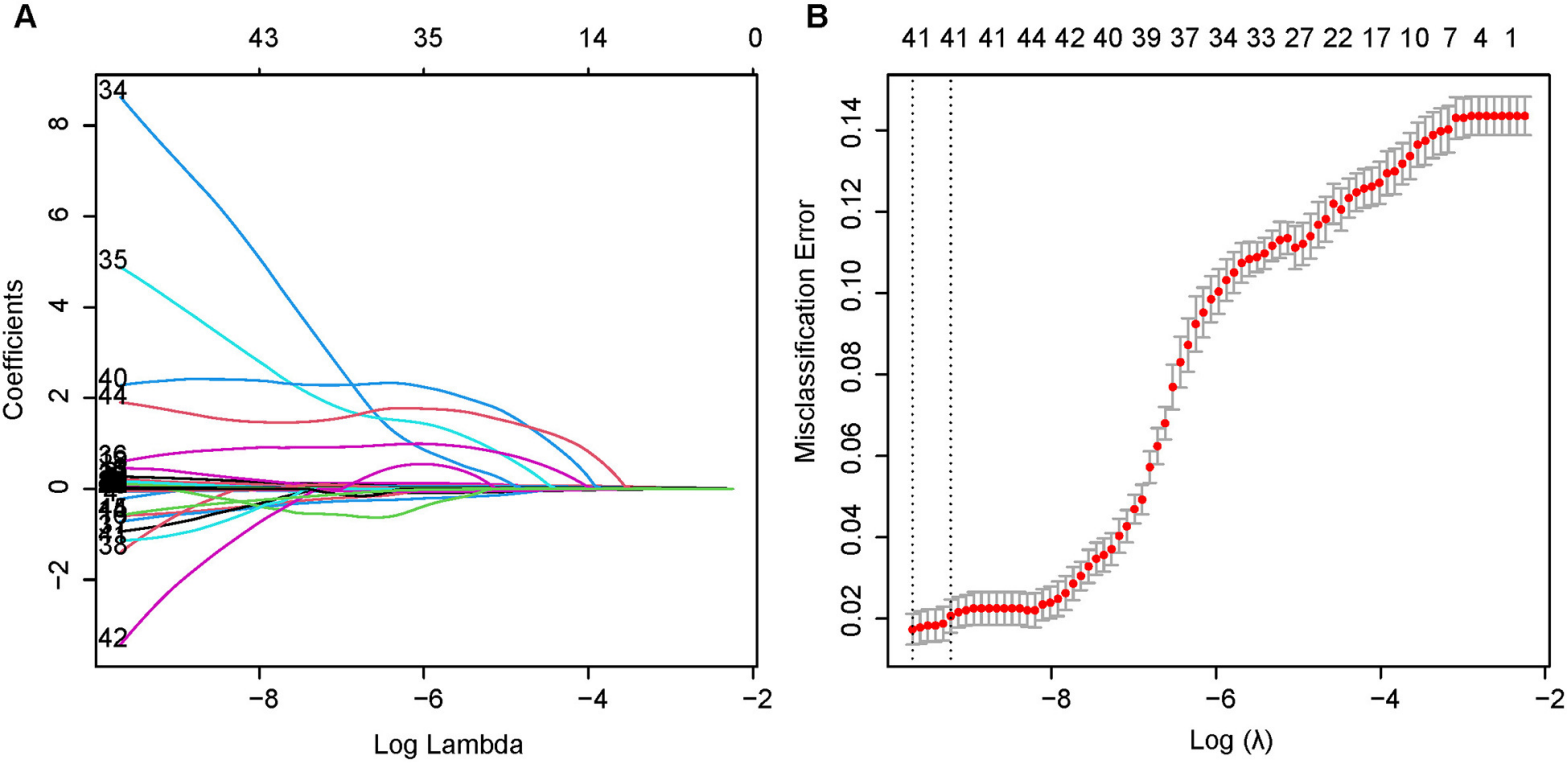
Least absolute shrinkage and selection operator (LASSO) binary logistic regression model for identifying independent risk factors for mortality in patients with SEMI within 28 days. **(A)** LASSO coefficient profiles of the radiomic features. Each colored line represents the coefficient of each feature. **(B)** Plot the results of cross-validation, and the red dots in the figure represent the target parameters corresponding to each lambda. The largest lambda value is chosen when the cross-validation error is within one standard error of the minimum.

Independent risk factors affecting mortality within 28 days in patients with ICU subendocardial infarction were determined by using least absolute value shrinkage and choice operator regression. Figure 2A,B illustrates the different mean square errors in the log(lambda) range. When the cross-validation error is less than the standard error of the minimum value, the largest lambda value is selected. Multiple logistic regression analyses were performed to determine age, GCS score, creatinine, hematocrit, hemoglobin, international normalized ratio, blood urea nitrogen level, urine output, heart rate, respiratory rate, saturation of peripheral oxygen, peripheral vascular disease, diabetes complications, and solid tumors as independent risk factors for mortality during patient hospitalization.

**Figure 3:**
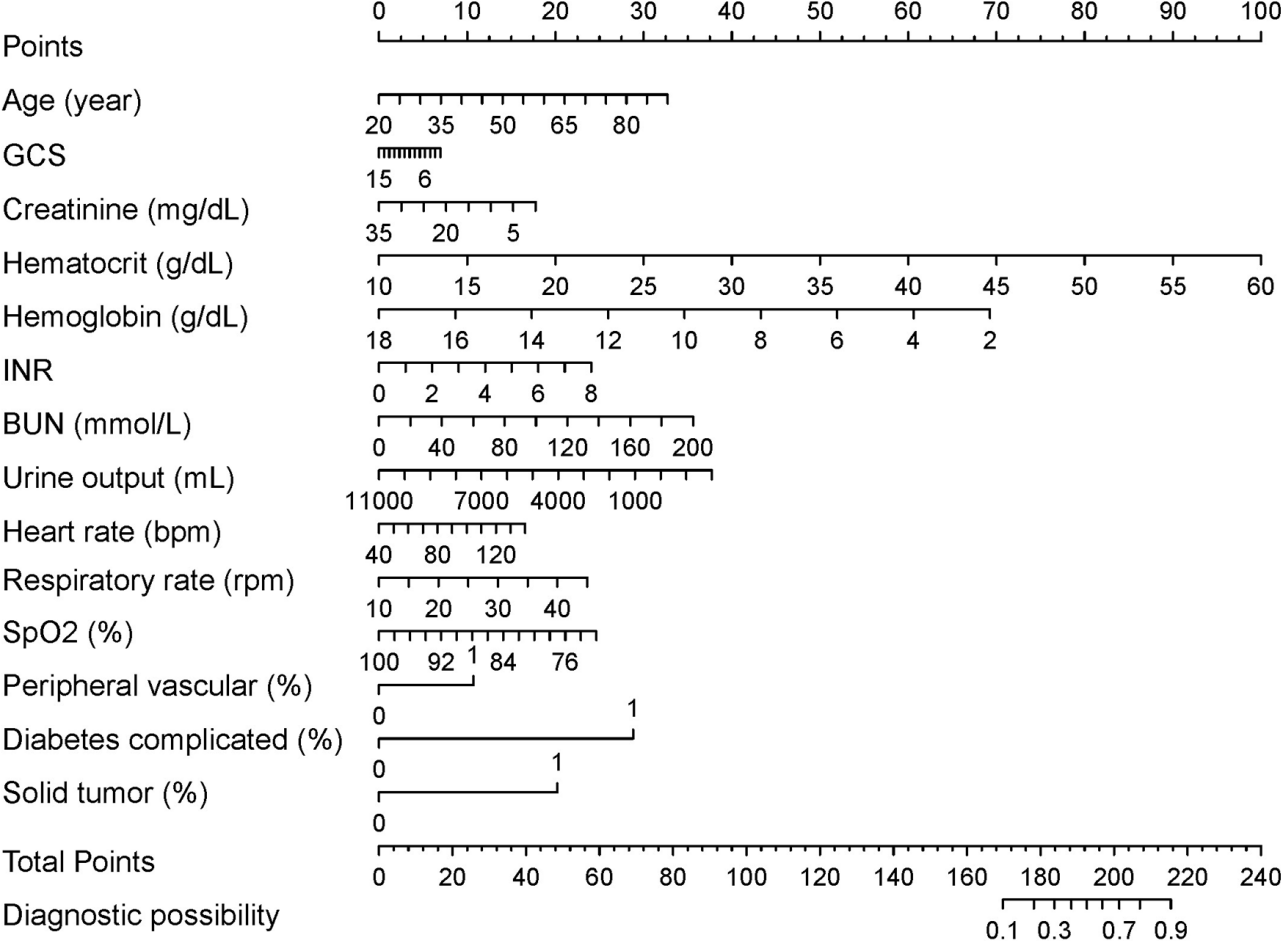
28-day mortality nomogram for subendocardial infarction. Nomogram included age, GCS score, creatinine, hematocrit, hemoglobin, international normalized ratio, blood urea nitrogen level, urine output, heart rate, respiratory rate, saturation of peripheral oxygen, peripheral vascular disease, diabetes complicated and solid tumor for predicting 28-day mortality after subendocardial infarction. The total point was calculated as the sum of the individual scores for each of the fourteen variables included in the nomogram. Patients were assessed based on each variable, and the total points were assigned according to the nomogram. Each variable's specific data were used to evaluate the patient, resulting in a total score derived from the nomogram. With this value, the risk of 28-day mortality could be predicted.

The risk of mortality within 28 days was 1.05 times higher in older patients than in younger patients (CI = 1.036–1.067). The risk of death was 1.25 times higher in patients with a higher hematocrit level at the first laboratory test than in patients with normal results (CI = 1.118–1.404). Patients with a higher respiratory rate at ICU entry had 1.05 times higher risk of death than patients with a normal outcome (CI = 1.012–1.086). The risk of mortality was 8.11 times higher (CI = 2.759–23.372) in patients with a solid tumor at the time of ICU entry than in normal patients, and 17.00 times higher (CI = 1.556–251.854) in patients with diabetes complications. Additionally, the international normalized ratio (OR = 1.241, CI = 1.015–1.508), blood urea nitrogen level (OR = 1.013, CI = 1.005–1.020), heart rate (OR = 1.014, CI = 1.003–1.025), and peripheral vascular disease (OR = 2.922, CI = 1.363–5.952) were also risk factors for 28-day mortality in patients with subendocardial infarction. The OR values of the creatinine level (OR = 0.870, CI = 0.768–0.973), hemoglobin level (OR = 0.573, CI = 0.414–0.790), urine output (OR = 0.999, CI = 0.999–1.000), saturation of peripheral oxygen (OR = 0.938, CI = 0.882–0.996) and GCS score (OR = 0.939, CI = 0.895–0.986) were all less than 1 and were therefore considered protective factors (Table 2). These results were utilized to construct a nomogram that predicted the risk of mortality within 28 days in patients with subendocardial infarction (Figure 3).

**Table 2:** Factors independently associated with 28-day mortality in subendocardial infarction patients.

Variables	OR	95% CI	*P*
Age (year)	1.0512	1.03639–1.06678	0***
GCS	0.9385	0.89509–0.98607	0.01*
Laboratory test			
Creatinine (mg/dl)	0.8695	0.76800–0.97315	0.02*
Hematocrit(g/dl)	1.2527	1.11842–1.40431	0***
Hemoglobin(g/dl)	0.5732	0.41449–0.79008	0***
INR	1.2408	1.01543–1.50816	0.03*
BUN (mmol/L)	1.0128	1.00528–1.02042	0***
Urine Output (ml)	0.9998	0.99962–0.99989	0***
Vital signs			
Heartrate (bpm)	1.0138	1.00268–1.02513	0.02*
Respiratory rate (rpm)	1.0482	1.01185–1.08573	0.01*
SpO_2_ (%)	0.9375	0.88191–0.99576	0.04*
Comorbidities			
Peripheral vascular (%)	2.9220	1.36320–5.95237	0***
Diabetes complicated (%)	16.9977	1.55640–251.85395	0.03*
Solid tumor (%)	8.1090	2.75856–23.37158	0***

* *P* < 0.05.

*** *P* < 0.001.

BUN, blood urea nitrogen; INR, international normalized ratio; SpO2, peripheral oxygen saturation.

### Nomogram validation

**Figure 4:**
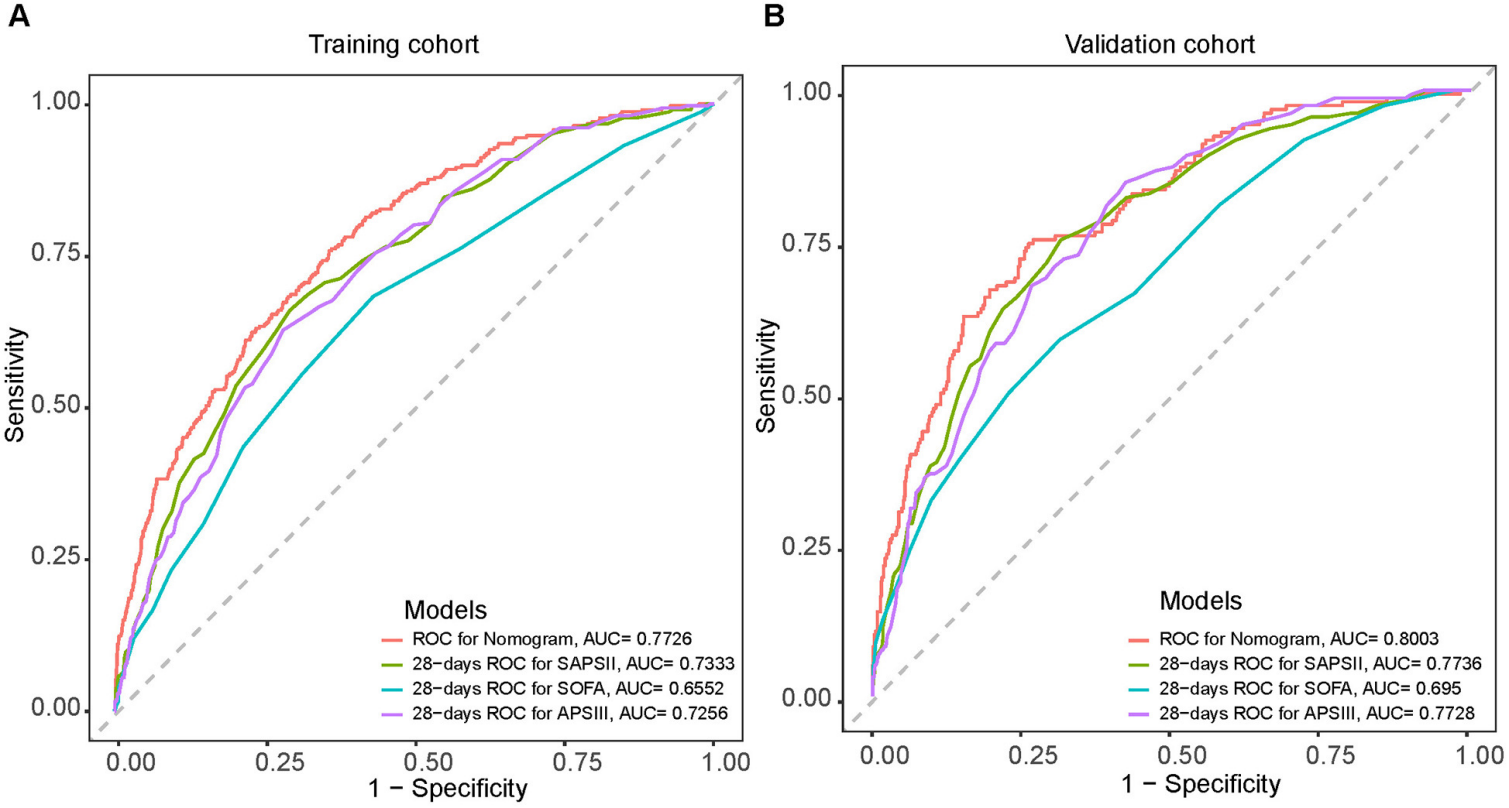
Receiver operating characteristic (ROC) curves for the SAPSII (green), APSIII (purple), SOFA (blue) and the nomogram (Red) in the training cohort **(A)** and the validation cohort **(B)**.

Harrell's consistency index (C-index) and ROC curves were employed to analyze the discriminative power of our nomogram. The C-index was 0.810 (95% CI = 0.830–0.790) and 0.807 (95% CI = 0.840–0.770) for the training and validation groups, respectively. The ROC curves in Figure 4A,B validate the overall predictive performance of the nomogram. The AUC of the nomogram in the training and validation groups is 0.7895 (95% CI = 0.764–0.815) and 0.8066 (95% CI = 0.772–0.841), respectively. Our nomogram model exhibited higher AUC values than the APSIII, SAPSII, and SOFA scoring system in both the training and validation groups. The optimal cutoff value of the nomogram was determined based on the Youden index, which was 0.150 in the training group, with specificity and sensitivity of 0.742 and 0.712, respectively. In the validation group, the optimal cutoff value of the nomogram was 0.113, with specificity and sensitivity of 0.589 and 0.873, respectively. The AUC values of the nomogram and other scoring systems were further compared using the Delong's test. In the training cohort, the AUC of the nomogram was significantly larger than that of APSIII (*Z* = 3.275, *P* = 0.001), SAPSII (*Z* = 2.361, *P* = 0.018), and SOFA (*Z* = 6.504, *P* = 0.001), suggesting that our model possesses discriminative ability superior to traditional scoring systems.

Compared to the traditional APSIII, SAPSII, and SOFA scoring systems, the NRI values for the nomogram were 0.224 (95% CI = 0.115–0.392), 0.211 (95% CI = 0.077–0.367), and 0.3811 (95% CI = 0.273–0.559) for the training group, respectively. It was 0.204 (95% CI = 0.066–0.491), 0.220 (95% CI = 0.035–0.471) and 0.367 (95% CI = 0.197–0.689) in the validation group, respectively. The corresponding IDI values for the training group were 0.057 (95% CI = 0.038–0.076), 0.047 (95% CI = 0.026–0.069), and 0.088 (95% CI = 0.067–0.108), respectively. For the validation group, the corresponding IDI values were 0.065 (95% CI = 0.032–0.099), 0.058 (95% CI = 0.023–0.093), and 0.105 (95% CI = 0.070–0.140). These values indicate that our model has excellent discriminative power comparable to currently widely used scoring systems.

**Figure 5:**
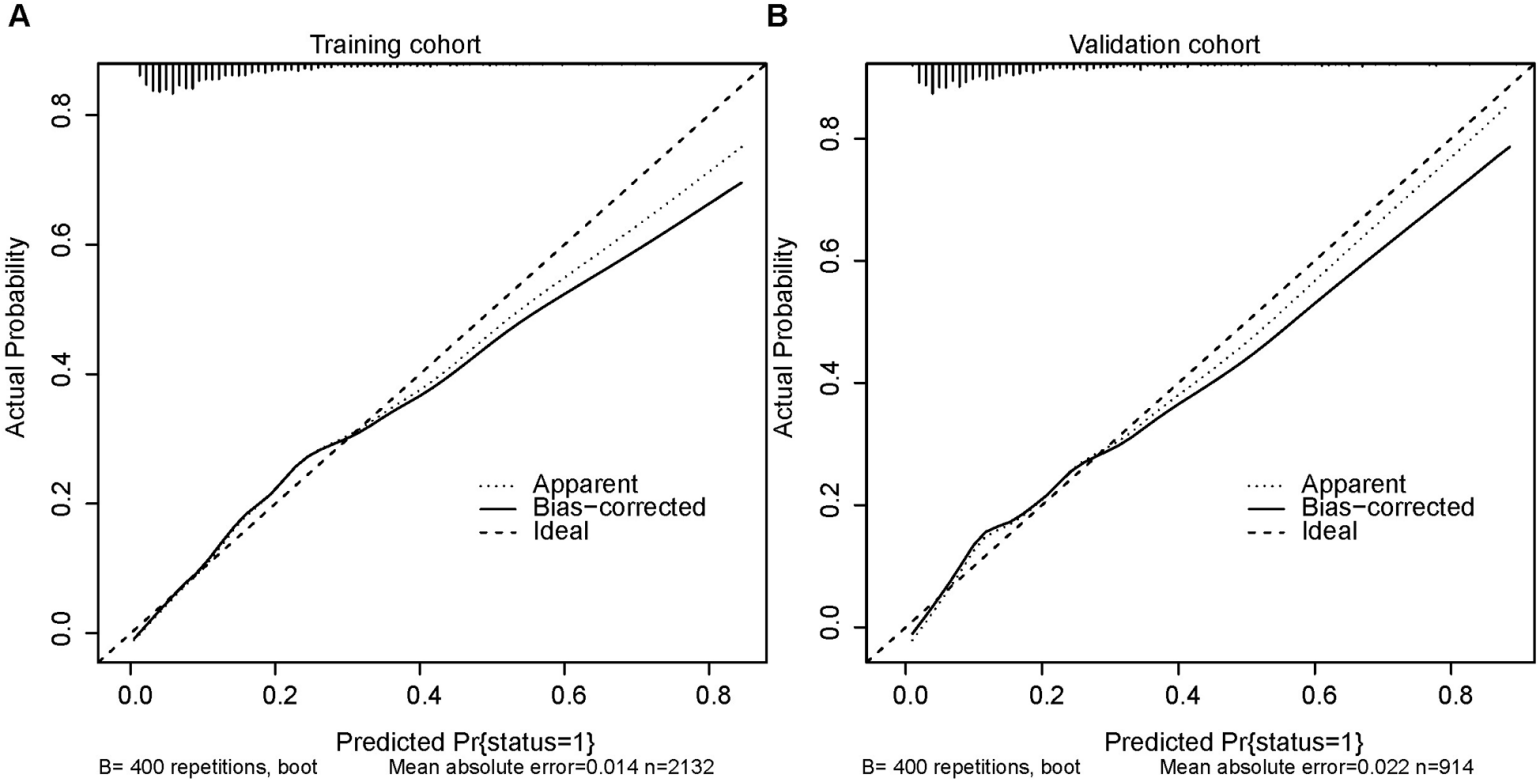
Calibration curves for the training cohort **(A)** and the validation cohort **(B)**.

**Figure 6:**
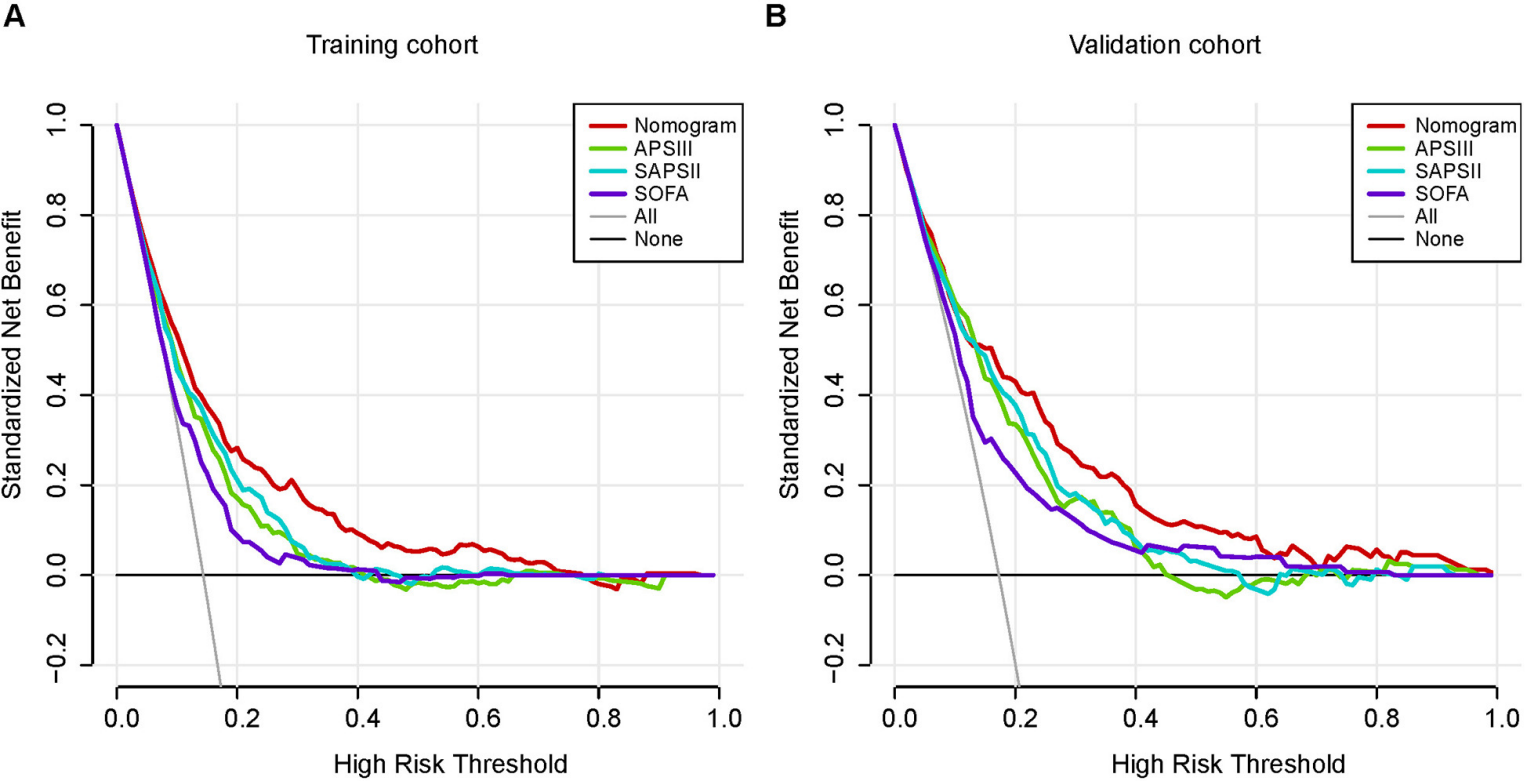
Decision-curve analysis of the training cohort **(A)** and the validation cohort **(B)**. Decision curve analysis depicts the clinical net benefit in pairwise comparisons across the different models. The red line indicates the nomogram, which is the model we built. The green line indicates APSIII scoring system, the blue line indicates SAPSII and the purple line indicates SOFA. Nomogram showed superior net benefit with a wider range of threshold probabilities compared with SAPSII, APSIII and SOFA.

Figure 5A,B depicts the calibration curves of the nomogram. It is evident from the figure that the calibration curves for both the training and validation groups closely align with the diagonal. There is no significant difference in the Hosmer-Lemeshow test results (training group: *χ*^2^ = 10.375, *P* = 0.240; validation group: *χ*^2^ = 14.018, *P* = 0.081). The corresponding DCA curves, comparing the nomogram with the APSIII, SAPSII, and SOFA scoring systems, are presented in Figure 6A,B. In both the training and validation groups, the net benefit of clinical interventions guided by our nomogram model exceeded those guided by the APSIII, SAPSII, and SOFA scoring systems when the threshold probability ranged from 0.2 to 0.7, demonstrating the clinical applicability of our nomogram.

## Discussion

The aim of our study was to investigate the relationship between factors and the risk of death in patients with subendocardial myocardial infarction and to develop an associated nomogram to predict the risk of mortality in these patients. We utilized LASSO and multivariate logistic regression to identify independent risk factors for mortality within 28 days in patients with subendocardial myocardial infarction, including age, GCS score, creatinine, hematocrit, hemoglobin, international normalized ratio, blood urea nitrogen level, urine output, heart rate, respiratory rate, saturation of peripheral oxygen, peripheral vascular disease, diabetes complications, and solid tumor. We constructed a nomogram model to assess the 28-day risk of mortality in subendocardial infarction patients based on these independent factors. Some indicators in the study suggest that the constructed nomogram exhibits good predictive performance.

Previous studies have indicated that patients with subendocardial infarction in ICU centers face a significant risk of death and further cardiac events (18). However, studies on the prognostic risk factors for patients with subendocardial myocardial infarction remain incomplete. Therefore, the development of a predictive model to forecast the risk of in-hospital mortality in these patients is highly warranted. Our finding that age is an independent risk factor for poor prognosis in patients with subendocardial myocardial infarction aligns with numerous prior studies (8). Age is widely acknowledged as a significant risk factor for subendocardial myocardial infarction (8, 19), with less than 10% of patients younger than 45 years of age experiencing myocardial infarction (20). The Glasgow Coma Scale (GCS) is a structured method for assessing the level of consciousness (21), applicable to all types of acute medical and trauma patients to objectively describe the degree of impaired consciousness. Our results demonstrate a significant correlation between low GCS scores and high rates of mortality and poor prognosis (22). Therefore, accurate clinical assessment necessitates comprehensive behavioral measures that rigorously and reliably classify patients based on their level of awareness, cognition, and functioning (22).

Elevated serum creatinine has been identified as a robust independent risk factor for death following myocardial infarction in previous studies (8, 23), consistent with our nomogram. Serum creatinine serves as a commonly used biomarker of renal function. Patients experiencing myocardial infarction alongside end-stage renal disease have been shown to have elevated mortality rates from cardiac causes and poor long-term survival (24). Thus, the predictive value of high creatinine levels for poor prognosis in acute myocardial infarction is typically closely linked to renal damage or dysfunction observed in patients with impaired cardiac function (23).

Our study indicates that hematocrit, hemoglobin, and INR are independent risk factors influencing poor prognosis in patients with subendocardial infarction. Hematocrit, the volume percent of red cells in blood, serves as a crucial determinant of blood viscosity (25). Elevated hematocrit levels may contribute to the development of myocardial infarction, in which a decrease in plasma volume is more common than an increase in red blood cell mass, suggesting that hemoconcentration may play a role in the etiology of myocardial infarction (26). One study found that lower hemoglobin levels were associated with a poor prognosis in patients with subendocardial infarction (27), which is consistent with our results. Lower hemoglobin levels upon admission to the hospital in patients with myocardial infarction causing a poor prognosis may be associated with relatively greater myocardial damage (28). The International Normalized Ratio (INR) is a standardized tool used to compare prothrombin time measurements with the international reference thromboplastin. Abnormally elevated INR is associated with severe clinical symptoms and increased mortality (29). Our study indicates that hemoglobin levels exhibit a J-shaped relationship with mortality risk in SEMI patients. The RCS analysis suggests that both excessively low and high hemoglobin levels may be associated with adverse outcomes. Specifically, a hemoglobin level below 11 g/dl has been widely recognized as a marker of anemia, which may contribute to poor cardiovascular prognosis due to reduced oxygen transport capacity, increased cardiac workload, and heightened risk of ischemia (30, 31). This aligns with prior research that demonstrated a significant association between lower hemoglobin levels and increased short-term mortality in acute myocardial infarction (27). Furthermore, significantly higher hemoglobin levels (>16 g/dl) have also been linked to increased cardiovascular mortality, likely due to elevated blood viscosity, increased shear stress, and a greater risk of thrombotic events (27, 32). These findings reinforce the importance of maintaining an optimal hemoglobin range for better prognosis in SEMI patients.

Patients with myocardial infarction may undergo rapid changes in renal function due to hemodynamic alterations and systemic inflammatory responses, leading to acute kidney injury (33). The development of acute renal dysfunction is closely linked to poor clinical outcomes in patients experiencing cardiogenic shock after myocardial infarction (34). Impaired renal function is notably characterized by the accumulation of nitrogen metabolism end products (urea and creatinine), decreased urine output, or both (33). Blood urea nitrogen (BUN) levels can demonstrate unfavorable acute hemodynamic and neurohumoral changes (35). Therefore, BUN levels, along with other renal function parameters, may serve as useful diagnostic tools in a multivariate risk assessment approach to cardiovascular disease (36). Elevated BUN levels have been independently shown to predict short- and intermediate-term mortality in patients with acute myocardial infarction (36, 37), which is consistent with our findings. Additionally, decreased urine output reflects the severity of acute kidney injury (38), which may also predict a poor prognosis in patients with subendocardial infarction in the intensive care unit, aligning with our results.

Elevated heart rate upon admission or during hospitalization in patients with myocardial infarction correlates significantly and independently with mortality (39), which supports our findings. Increased heart rate may directly impact cardiovascular risk through several hypothesized mechanisms, the most prominent being its association with heightened myocardial oxygen demand (40). While tachycardia in heart failure might serve as a compensatory response to some extent (40), this isn't the case during myocardial infarction. Here, a high heart rate leads to elevated myocardial oxygen consumption and reduced myocardial perfusion, potentially worsening myocardial ischemia and resulting in a more severe infarction (41). Substantial evidence indicates that abnormal respiratory responses in heart disease serve as indicators of poor prognosis (42, 43), consistent with our findings.

Acute myocardial infarction primarily results from a mismatch between myocardial blood oxygen supply and demand, leading to ischemia and eventual cell death (44). Consistent with our findings, hypoxia (low oxygen levels) have been identified as an independent marker of poor prognosis in myocardial infarction (44, 45). In a related study, it was observed that nonsurvivors exhibited lower cardiac index, hemoglobin concentration, and oxygen saturation, along with significantly higher oxygen demand compared to survivors of acute myocardial infarction (46). This observation may be attributed to an impaired oxygen delivery system in patients with myocardial infarction. Additionally, patients with low oxygen saturation face an increased risk of heart failure and have a notably high mortality rate (47), highlighting the importance of emphasizing this indicator.

Furthermore, we have found that certain comorbidities increase the risk of adverse outcomes in subendocardial infarction. Peripheral vascular disease affects blood vessels outside the brain and heart, often resulting from narrowed or blocked blood vessels and atherosclerosis (48). The presence of comorbid peripheral vascular disease in patients experiencing acute MI increases the risk of in-hospital mortality (49). This may be linked to factors such as extensive coronary artery disease and inflammatory response (50). Diabetes mellitus has consistently been shown in numerous studies to be a risk factor for poor prognosis following myocardial infarction (51). Factors contributing to the elevated incidence of poor outcomes in diabetic patients may include an accelerated atherosclerotic process, left ventricular systolic and diastolic dysfunction, metabolic disorders leading to myocardial dysfunction, and sympatho-vagal imbalance (51, 52). In addition, our study has identified solid tumor as a risk factor for adverse outcomes in patients with subendocardial infarction. There is evidence suggesting that myocardial infarction and cancer share similar molecular pathways in disease onset and progression (53). Mechanisms such as inflammation, neurohormonal activation, oxidative stress, and immune system dysfunction are common to both conditions (54). Therefore, special attention should be given to therapeutic strategies and prognostic tools for patients with comorbidities in the clinical setting.

This study utilized a substantial volume of clinical data from the MIMIC-III public database to construct a clinical prediction model with enhanced predictive performance, which could provide valuable insights for clinical practice. The accuracy and reliability of the data in the MIMIC-III database have been well established. The large dataset used in this study reduces the likelihood of random errors, enhancing the reliability of the results. Additionally, the patient information in this database is anonymized, which helps to minimize potential biases in the sampled patient population. However, our study has some limitations. Firstly, the database used in this study includes data from only one medical center. Despite the large number of patients included, there is still a risk of selection bias. More multicenter and prospective studies are required in the future to confirm these findings. Secondly, a significant limitation of this study is the exclusion of some patients due to a high rate of missing data, which might have introduced bias into the results. Lastly, this study lacks external validation to confirm its utility. Future studies with external validation using additional data sources will be crucial for improving the credibility and generalizability of the results.

## Conclusion

Our study identifies independent risk factors influencing the 28-day adverse prognosis of patients with subendocardial infarction in ICU and establishes a nomogram based on these factors. This nomogram can be utilized to predict mortality in patients with subendocardial infarction in the ICU. These findings provide valuable insights for clinical management and are expected to enhance the prognosis and treatment of patients with subendocardial infarction.

## Data Availability

Publicly available datasets were analyzed in this study. This data can be found here: Publicly available datasets were analyzed in this study. This data can be found here: MIMIC-III: https://www.physionet.org/content/mimiciii/1.4/.
